# Supplementary material for: An extremely fast neural mechanism to detect emotional visual stimuli: A two-experiment study
Source: PLoS One. 2024 Jun 21;19(6):e0299677. doi: 10.1371/journal.pone.0299677 (PMC11192326; doi:10.1371/journal.pone.0299677)
Supplement: Attachment — Submitted filename: ResponseToReviewers.docx [file pone.0299677.s001.docx]

**RESPONSE TO REVIEWERS**

Response to Reviewer 1 🡪 p.1

Response to Reviewer 2 🡪 p. 6

Appendix 🡪 p. 8

References 🡪 p. 10

**REVIEWER 1**

**We sincerely thank Reviewer 1 for his/her feedback and for the time and thorough review of the manuscript and his/her relevant and constructive suggestions. This paper is much improved thanks to his/her helpful comments. Comments and actions to each suggestion are described next. Original reviewer comments in regular typeface, responses in boldface. Please note that the pages mentioned here are as appear in the actual paper, not in the PDF viewer used to read it.**

I have two major reservation, one about the organization of the manuscript and the other about the complicated data analysis.

A) In my opinion it is not necessary and even disadvantageous to divide the manuscript according to “the two experiments”. These are not really two experiments, but one experiment with constant methodology and two participant groups submitted to variants of a stimulation procedure. You can avoid all the redundancy by describing common methodology in one paragraph; listing consecutive results and writing one Discussion instead of three of them.

**We appreciate this suggestion to simplify the structure of the paper. We have reorganized the paper following this recommendation by Reviewer 1.**

B) Concerns regarding analytical approach
1) I am not at all convinced that PCA approach was really justified for this particular experiment. Data reduction was indicated as a reason to use it, but data reduction could have been done in a classical way by choosing one or few electrodes of interest in the occipital area and averaging them, just like a time-window of interest was selected on the timescale. Actually, you do it for the figures (the waveforms called “meta-averaged recordings”). PCA was applied to amplitude values extracted from all 64 electrodes, while the N40 is expected to be detected in a few of them. It is a waste of a method power – PCA was working on the variance not related to the question of this experiment.
If there is any other benefit from PCA (above data reduction) it should be clearly indicated.

**We understand Reviewer 1’s concerns and the need to explain (also in the paper) why PCA is preferable over traditional methodologies. First of all, we would like to indicate that we have analyzed our data following the traditional methodology as suggested by Reviewer 1 (please see the Appendix to this response to reviewers), and to make it clear that the significant spiders>wheels effects continue to be significant regarding the N40-P80 (Exp. 1) and N40-P100 (Exp. 2) peak-to-peak amplitudes. However, we would like to explain why PCA-based analyses are preferable over traditional ones (e.g., Chapman & McCrary, 2005; Coles et al., 1986; Dien, 2012; Donchin & Heffley, 1978) and why we consider the quality of the paper benefits from keeping them (however, if still considered necessary after this explanation, we would proceed to change to traditional).**

**On one hand, it is important to note that the contribution of each scalp location (hereafter “electrode” for simplification) to a particular ERP component 1) is different from the contribution of its neighbor electrodes and 2) is not an all-or-nothing involvement. In other words, each electrode has a different weight (or involvement) in a particular component, and even those relatively far from the “peak electrode” (i.e., the most contributing electrode) have a certain, non-zero, involvement. Traditional analyses select a subset of electrodes and manage them with the same weight as if they are equally involved in the component (please note that this is not correct from a physiological point of view) and those electrodes out of the subset or region selected are ignored or conceptualized as zero-contributors (which is also physiologically incorrect). Instead, sPCA defines a weight for each electrode, which is different from the rest (slightly in the case of neighbor electrodes, more pronounced in the case of further electrodes).**

**Additionally, these weights are data-driven, physiologically based: regions (or *spatial factors* in PCA nomenclature) are defined by those electrodes that covary in the specific time window analyzed or, in other words, by those electrodes placed over the neural areas involved in the particular neural process occurring in that time window. As a result, PCA- (or physiologically-) based regions are usually relatively irregular or non-geometrical. Instead, traditional analyses usually define regular or geometrical regions which, from a physiological point of view, are far from being realistic.**

**Moreover, neural processes concurring at a particular moment spatially overlap. The traditional methodology (in the space domain) is based on the single topographic map provided by the grand averages for a particular time point or window, and in this single map all the neural processes (all spatial foci) compete, showing an electrical balance of all of them at each electrode. In graphical terms, this causes distortions of foci (shrinkages, displacements, etc.) and some foci may be even neutralized and not appreciable in the grand average-derived single scalp topography. Also in graphical terms, sPCA “separates” each neural process in different maps which draw its topography free of the influence of other adjacent or subjacent processes occurring in the same time window (an example of disentangling ERP components presenting temporospatial overlapping: Haese & Czernochowski, D., 2022).**

**And finally, but not less important, the projection brain-scalp is not direct. Thus, for example, occipital cortex activation may not be reflected in occipital scalp maxima (this is just what happens with C1, the earliest visual ERP component whose scalp topography has been described so far: despite its origin is V1, its peak amplitudes are observed at central-parietal scalp regions: Capilla, Melcón, Kessel, Calderón, Pazo-Álvarez & Carretié, 2016; di Russo, Martínez, A., Sereno, M. I., Pitzalis, S., & Hillyard, 2002). This point is important to be taken into account in the region-of-interest (ROI) definition based on previous literature, the second traditional criterium, along with the topographic map of grand averages, to define ROIs.**

**We have summarized this information in the paper so the advantages of PCA beyond data reduction are now explained (please see next point: this new text also includes information discussed there).**

2) The other point is the description of the analysis and reporting of the results. I admit that I am not a hard-core neuroinformatics specialist (like most of putative readers of this report), and I had a difficulty understanding how the data was fed into PCA. There was only one analyzed measure (i.e. amplitude); it is not obvious how were defined the variables fed to PCA – electrodes? how were defined the samples? participants? How were the emotional conditions taken to account? were they fed as independent samples in one analysis (i.e. N40_sub1_wheel; N40_sub1_spider; N40_sub2_wheel; N40_sub2_spider; …)? If so, I would be afraid that between-subject variance could be much higher than between-condition variance, again compromising the merit of PCA analysis. Components were selected based on the scree plot – but what was the criterion/ cut off eigenvalue for principal components (PC) acceptance? Only one (out of five substantial PCs) was chosen for further analysis based on the topography i.e. high loading scores into occipital electrodes – what was the level of variance explained by these PCs?

**We understand that information on how sPCA is computed was scarce in the first version of the paper and the readers will acknowledge more details. Indeed, in sPCA (and, in general, in factorial analyses), variables are electrodes and cases are participants x conditions. In other words, a single sPCA is carried out for the whole data matrix corresponding to a time point (or to a time window average). Basically, those electrodes covariating to a greater extent across participants and conditions have more weight in a particular spatial factor. The whole spatial factor (or region, but it is important to remember that all electrodes contribute to each spatial factor, albeit with different weights) is “summarized” in the spatial factor score, a single parameter per participant and condition that is basically the summatory (across electrodes) of weight x voltage: those electrodes more involved in the spatial factor (e.g., frontal electrodes in a frontally-distributed factor) or, in other words, presenting the highest weights (close to 1), will contribute much more to that spatial factor than those electrodes not involved and therefore presenting close-to-zero weights (quasi-zero x any voltage that electrode may present yields quasi-zero, not contributing in the summatory or spatial factor score). This spatial factor score, one per participant and condition per spatial factor (or region), is then linearly related to voltage, and may be submitted to any statistical test, but with the advantages mentioned in the previous point.**

**As regards the level of variance explained by each of the selected spatial factors, it is important to indicate that those covering more scalp surface (affecting more electrodes) explain more variance (given that sPCA variables are the electrodes), and those more circumscribed to smaller scalp regions explain less. Please note that in ERP research this is not crucial information, since the relevant spatial factor from a physiological point of view is not always the most extensive. For example, in experiments on facial processing, N170 relevant factor scores (i.e., those showing maxima at PO7 / PO8) are relatively small in terms of scalp surface (e.g., Giménez-Fernández et al., 2020) and explain less variance than other spatial factors, more extensive, that usually appear at the same time window (e.g., a fronto-central huge factor usually appears) but are not directly related to face processing. Something similar can be said about the N40 component in our study.**

**We have summarized this information, as well as the advantages discussed in the previous point, in the new version of the paper (p. 13, l.20 to p.14, l. 14):**

**“Principal component analysis has been long defended as a preferable methodology to detect and quantify ERP components over traditional methodologies (e.g., Chapman & McCrary, 2005; Coles et al., 1986; Dien, 2012; Donchin & Heffley, 1978). In the space domain (sPCA), the main advantage of PCA over classical procedures based on visual inspection of topographies to define regions of interest is that it presents each ERP component separately and with its ‘clean’ shape, extracting and quantifying it free of the influences of adjacent or subjacent components. Indeed, several neural processes (and hence, several electrical signals) may concur at any given moment, and the recording at any scalp location at that moment is the electrical balance of these different neural processes. Such recording can stymie visual inspection. Spatial PCA (sPCA), in which variables are the electrodes and cases are participants x conditions, separates ERP components along space, each spatial factor ideally reflecting one of the concurrent neural processes occurring at any given moment or temporal interval. Additionally, sPCA provides a reliable division of scalp into different recording regions. Basically, each region or spatial factor (SF) is formed with the scalp points where recordings tend to covary. As a result, the shape of the sPCA-configured regions is functionally based, and scarcely resembles the shape of the geometrically configured regions usually defined by traditional procedures. The spatial factor score, the sPCA-derived single parameter (per participant and condition) in which each SF is quantified, “summarizes” the behavior of the whole set of electrodes it involves (with different weights) and is linearly related to original amplitudes. ”**

Other comments

Introduction
page 4 line 20 “Nonfacial, consciously perceived emotional stimuli have not been explored in this respect.”
ERP difference in 50-100 ms window was shown for emotional words:
Rellecke, J., Palazova, M., Sommer, W., & Schacht, A. (2011). On the automaticity of emotion processing in words and faces: event-related brain potentials evidence from a superficial task. Brain and Cognition, 77(1), 23–32. 

**We thank Reviewer 1 for this information. We were not aware of this report. We now mention it in the new version of the paper (p. 4, l. 20-23).**

page 4 line 22
“Although less frequently, visual ERP components before C1 have also been reported “
It is worth noting that such waves are detected during standard clinical VEP recording with the use of flash stimuli which evoke series of waves starting as early as 30 ms.
Odom, J. V., Bach, M., Brigell, M., Holder, G. E., McCulloch, D. L., Mizota, A., Tormene, A. P., & International Society for Clinical Electrophysiology of Vision. (2016). ISCEV standard for clinical visual evoked potentials: (2016 update). Documenta Ophthalmologica. Advances in Ophthalmology, 133(1), 1–9.

**We completely agree it is important to refer to the ISCEV standard and to the idea that pre-C1 components are well-established in clinical practice. Thank you for this suggestion. We now include this idea in the introduction (p. 5, l. 1-3).**

Methods

What was the software used for stimuli presentation? Haw was it communicated with EEG recording system.

**The software was Psychtoolbox 3, which works under Matlab. The communication between the stimulation PC and the recording system (Biosemi) is via optic fiber as also implemented by Biosemi. We specify this information in the new version of the paper.**

**Regarding the critical issue of the inevitable lag that every stimulation system presents between the stimulus onset command (PC) and its actual onset in the screen, we indicated in the first version of the paper (and also in the new one, but we have moved it to “Stimuli” section): “the inevitable lag between the marks signaling stimuli onsets (or ‘triggers’) in EEG recordings and its actual onset on the screen was measured employing a photoelectric sensor as described in https://www.youtube.com/watch?v=0BPwcciq8u8 and corrected during pre-processing”.**

**These changes appear in p. 7, l. 7-9.**

You declare to record 64 channels according to international 10-20 system, however there is no single one and only electrode pattern for 64 channels. Electrode map could be added in supplementary files.

**Done. The text now refers to Supplemental material (p. 10, l. 15-16) and the map is uploaded there.**

N40 detection: “the occurrence of N40 was confirmed when at least two neighbor channels (within the relevant scalp region, i.e., the posterior hemiscalp) presented at least two consecutive voltage points whose amplitude was beyond ±1.5 times the standard deviation of the corresponding baseline.”
The +/- sign is confusing – in a search for a negative wave, you accepted also positive deviations from the baseline level?

**Since early ERP components may vary their polarity as a function of the spatial location where the stimulus appears (e.g., Capilla et al., 2016), we were looking for any deflection (positive or negative) meeting those SD criteria (we would have termed NP40 instead of N40 if it was positive for some stimulus location/s). However, as indicated in the paper, no deflections in the 30-50 ms window, neither positive or negative, were found for stimuli in the periphery.**

p11, line 18 “... eliciting an increase of absolute amplitude in both of them, as is apparently the case here (Figure 2).”
No amplitude increases are evidenced in figure 2. There is only one trace with hardly visible N40.

**There was an error in this respect in the first version of the paper, we should have written “Figure 3”, the figure in which a global increase in N40-P80 amplitude is observed in response to spiders. However, due to the restructuration of the paper mentioned in point A, Figure 3 in the first version of the paper is now Figure 4 (and vice versa): please see Table 1. Consequently, and to avoid citing Figure 4 too early, that part of the sentence, which is not essential at this point, has been deleted (the sentence now ends “…absolute amplitude in both of them.”). This sentence is now in p.13, l.10.**

| **First version of the paper** | **New version** |
| --- | --- |
| Figure 1 🡪 | Figure 1 |
| Figure 2 🡪 | Figure 2 |
| Figure 3 🡪 | Figure 4 |
| Figure 4 🡪 | Figure 3 |
| Figure 5 🡪 | Figure 5 |

**Table 1. Change in the order of some figures due to restructuration of the paper**

In this respect – have you tried setting logarithmic Y scale to highlight low values? Also, while I appreciate that presentation of ERP baseline is a good practice, in case of such a short window as you analyze and draw, baseline could be reduced (to <=25 ms) for enhanced visibility of post stimulus waves. Longer sweeps can be available in supplementary materials.

**We appreciate these suggestions. We have reduced 50% the baseline from (100 to 50ms) in all figures while uploading the grand averages with the whole baseline to supplemental material. We have also modified the vertical scale of Figures 2 to 5 (but still linear): the original scale had some 2 µV margin that grand average plots did not occupy. These changes make the plots vertically larger and enhance the visibility of N40 even in Figure 2 (where it was indeed less noticeable; it was more clearly visible in the rest of the figures).**

In Figure 3 (and 5) we see a corridor around VEP lines – what it is? why the same variance measure is not plotted in figures 2 (and 4)?

**It represents standard error of means (SEM); we failed to indicate this in figure legends, thank you for pointing this out. Figure legends include this information now. We have also added the SEM corridor in figures 2 and 4 (2 and 3 in the new version of the paper: Table 1).**

Why different electrodes are chosen if Fig 2 and Fig 3 for VEP averaging (“meta-averaging”)? For both cases they were chosen from the same PCA map, they should be the same.

**Actually, they illustrate different stages in the analytical path. Initially, grand averages from an apriori, literature-based, selection of electrodes were computed to define the windows of interest (WOIs) for analyzing N40 and P80 (in Experiment 1; N40 and P100 in Experiment 2). This initial stage of WOI definition is represented in Figure 2 (and Figure 3 in the case of Experiment 2), which also pretends to illustrate the subsequent outputs (in later stages) of this initial phase: sPCA and source location. sPCA is, therefore, an output of this initial phase, and was subsequently carried out on the WOIs defined in that initial step. On the other hand, Figures 4 and 5 (as numbered in the new version of the paper: Table 1) are “post-sPCA”, and represent the sPCA-derived results and a selection of data-driven electrodes (i.e., sPCA maxima) for graphical purposes only (statistical analyses were carried out on the whole spatial factors). We understand this was not clear in the first version of the paper, so we have implemented several changes in different parts of the paper to explain this more clearly, including figure legends.**

Page 19, line 12 “...one of her parents…” you should not disclose the sex of this participant, “...one of the parents…” would be better.

**Absolutely. Thank you for this. Done (p. 6, l. 21).**

Figures are informative but fig. 2-5 are drawn with too thin lines and with too small elements. They are unreadable when scaled to print size (at 600 DPI). Figures need thicker lines, bigger head plots, bigger fonts. On the other hand, some descriptions are proportionally huge (e.g. UL, LL, etc) and panel outlines very thick (~1 mm thick). Only the wave names have reasonable sizes.

**Following Reviewer 1 suggestions, we have changed the size of different elements of Figures 2 to 5: grand average plots are now thicker, as well as the grids inside those grand averages; lines in the legends are also thicker, and panel outlines have been reduced (up to a minimum which makes the code color -blue for N40, green for P80, grey for N40-P80 peak to peak amplitude- still visible), and huge descriptions (UL, LL, etc.) have been also reduced, following Reviewer 1 suggestions. The smallest labels, such as “peak to peak amplitudes” in new Figures 2 and 3, are bigger now. Additionally, we have reoriented the most complex Figure (#2) in the page from vertical to horizontal orientation to avoid conversion to PDF during uploading to PLoS One platform excesively reduces its quality and size.**

**REVIEWER 2**

The paper titled "An extremely fast neural mechanism to detect emotional visual stimuli: A two-experiment study" investigates the neural processes underlying the rapid detection of emotional visual stimuli. The authors present a series of experiments using event-related potentials (ERPs) to explore the early neural responses to emotionally negative (spiders) and neutral (wheels) stimuli. The study addresses an important and relatively unexplored area in the field of emotion processing, focusing on the rapid neural mechanisms involved in the detection of emotional visual stimuli. The paper is well-structured, with clear explanations of the methodology and results.

**We wish to thank Reviewer 2 for his/her positive comments and helpful suggestions for preparing an improved version of the manuscript. Please see below some considerations and actions derived from them. Original reviewer comments in regular typeface, responses in boldface. Please note that the pages mentioned here are as appear in the actual paper, not in the PDF viewer used to read it.**

However, there is room for improvement in terms of a more comprehensive discussion of limitations, broader theoretical implications, and potential individual differences:
1. The study's focus on a specific type of emotional stimulus (spiders) and a neutral control (wheels) may limit the generalizability of the findings to other types of emotional visual stimuli. In addition, the lack of a direct comparison with other emotional stimuli (e.g., positive or mixed emotions) leaves open questions about the specificity of the observed neural responses.

**We agree this is a necessary future step in this line of research and we develop this idea in the Discussion of this new version of the paper (from p. 27, l. 4-7).**

2. The study does not address potential individual differences in neural responses, which could be important for understanding variability in emotional processing.

**Thank you for this comment. Indeed, individual differences are also a natural further step in this line of research. We have added the following text to the discussion section:**

**“Relatedly, exploring individual differences is of great interest given that the activity of early evaluation structures is modulated by the individual experience thanks to the feedback they receive from other brain areas. For example, top-down modulation of visual cortices from evaluative structures higher in the hierarchy, such as the ventral prefrontal cortex (Bar et al., 2006), or of the visual thalamus from the visual cortex (Briggs & Usrey, 2008), plays a critical role in the activity of the initial evaluators.” (p. 27, l. 7-14).**

3. The paper could have included a discussion on the potential practical applications of the findings, such as their relevance to clinical populations or their integration into computational models of emotion processing.

These questions can be discussed in more detail during the discussion section.

**This is an interesting issue that was absent in the previous version of the paper. We now include a discussion on this in the passage where several structures, such as the visual thalamus and the superior colliculi, are described as candidates to be initial evaluation structures, given their reported link to certain affective disorders. The new insertion is as follows:**

**“Importantly, these structures modulate defensive behavior in response to visual stimuli in rodents (visual thalamus: Salay & Huberman, 2021; superior colliculus: Muthuraju et al., 2016). Concerning this, their abnormal activity has been proposed to be linked to affective problems such as anxiety or phobias in these same studies, pointing to their crucial role also at the clinical level.” (p. 26, l. 5-9).**

**APPENDIX**

**As indicated in response to Reviewer 1, we have carried out traditional analyses in the same time windows described in the paper for N40-P80/P100, but introducing direct voltages from critical electrodes according to previous literature. In this respect, please note that the earliest visual ERP component whose topography has been described is C1 (assimilable to P80), presents its maxima at midline central-parietal regions (Capilla, Melcón, Kessel, Calderón, Pazo-Álvarez & Carretié, 2016; di Russo, Martínez, A., Sereno, M. I., Pitzalis, S., & Hillyard, 2002). Electrodes selected for traditional analyses have been, therefore, C1, Cz, C2, CPz.**

**Experiment 1**

N40-P80 peak to peak amplitude for stimuli at fixation, same temporal windows as described in the paper (N40: 36-42ms, P80: 74-86ms). Results of repeated measures ANOVA on factors Electrode (see first paragraph) x Stimulus (Spiders, Wheels) and descriptive plots:

| **Within Subjects Effects** | | | | | | | | | | | | | |
| --- | --- | --- | --- | --- | --- | --- | --- | --- | --- | --- | --- | --- | --- |
| **Cases** | | **Sum of Squares** | | **df** | | **Mean Square** | | **F** | | **p** | | **η²_p_** | |
| Electrode |  | 12.445 | ᵃ | 3 | ᵃ | 4.148 | ᵃ | 15.159 | ᵃ | < .001 | ᵃ | 0.302 |  |
| Residuals |  | 28.734 |  | 105 |  | 0.274 |  |  |  |  |  |  |  |
| Stimulus |  | 16.160 |  | 1 |  | 16.160 |  | 6.000 |  | 0.019 |  | 0.146 |  |
| Residuals |  | 94.267 |  | 35 |  | 2.693 |  |  |  |  |  |  |  |
| Electrode ✻ Stimulus |  | 0.405 | ᵃ | 3 | ᵃ | 0.135 | ᵃ | 1.765 | ᵃ | 0.158 | ᵃ | 0.048 |  |
| Residuals |  | 8.031 |  | 105 |  | 0.076 |  |  |  |  |  |  |  |
|  | | | | | | | | | | | | | |
| *Note.*  Type III Sum of Squares | | | | | | | | | | | | | |
| ᵃ Mauchly's test of sphericity indicates that the assumption of sphericity is violated (p < .05). | | | | | | | | | | | | | |
|  | | | | | | | | | | | | | |

**Descriptive plots on N40-P80 amplitude as a function of factor Stimulus (Experiment 1). Error bars represent the standard error of means.**

**Experiment 2**

N40-P100 peak to peak amplitude for stimuli at fixation, same temporal windows as described in the paper (N40: 42-48 ms, P100: 99-111 ms). Results of repeated measures ANOVA on factors Electrode (see first paragraph) x Stimulus (Spiders, Wheels). and descriptive plots:

| **Within Subjects Effects** | | | | | | | | | | | | | |
| --- | --- | --- | --- | --- | --- | --- | --- | --- | --- | --- | --- | --- | --- |
| **Cases** | | **Sum of Squares** | | **df** | | **Mean Square** | | **F** | | **p** | | **η²_p_** | |
| Electrode |  | 46.913 | ᵃ | 3 | ᵃ | 15.638 | ᵃ | 50.937 | ᵃ | < .001 | ᵃ | 0.600 |  |
| Residuals |  | 31.314 |  | 102 |  | 0.307 |  |  |  |  |  |  |  |
| Stimulus |  | 18.054 |  | 1 |  | 18.054 |  | 7.897 |  | 0.008 |  | 0.188 |  |
| Residuals |  | 77.727 |  | 34 |  | 2.286 |  |  |  |  |  |  |  |
| Electrode ✻ Stimulus |  | 0.068 | ᵃ | 3 | ᵃ | 0.023 | ᵃ | 0.617 | ᵃ | 0.605 | ᵃ | 0.018 |  |
| Residuals |  | 3.769 |  | 102 |  | 0.037 |  |  |  |  |  |  |  |
|  | | | | | | | | | | | | | |
| *Note.*  Type III Sum of Squares | | | | | | | | | | | | | |
| ᵃ Mauchly's test of sphericity indicates that the assumption of sphericity is violated (p < .05). | | | | | | | | | | | | | |

**Descriptive plots on N40-P100 amplitude as a function of factor Stimulus (Experiment 2). Error bars represent the standard error of means.**

**REFERENCES CITED IN THIS RESPONSE TO REVIEWERS**

Capilla A, Melcón M, Kessel D, Calderón R, Pazo-Álvarez P, Carretié L. Retinotopic mapping of visual event-related potentials. Biol Psychol. 2016;118:114-125.

Chapman, R. M., & McCrary, J. W. (1995). EP component identification and measurement by principal components analysis. Brain and Cognition, 27, 288–310.

Coles, M. G. H., Gratton, G., Kramer, A. F., & Miller, G. A. (1986). Principles of signal acquisition and analysis. In M. G. H. Coles, E. Donchin, & S. W. Porges (Eds.), Psychophysiology: Systems, processes and applications (pp.183–221). Amsterdam: Elsevier.

Di Russo, F., Martínez, A., Sereno, M. I., Pitzalis, S., & Hillyard, S. A. (2002). Cortical sources of the early components of the visual evoked potential. Human brain mapping, 15(2), 95-111.

Dien, J. (2012). Applying Principal Components Analysis to Event-Related Potentials: A Tutorial. Developmental Neuropsychology, 37(6), 497–517.

Donchin, E., & Heffley, E. F. (1978). Multivariate analysis of event-related potential data: A tutorial review. In D. Otto (Ed.), Multidisciplinary perspectives in event-related brain potential research (pp. 555–572). Washington, DC: USA: Government Printing Office.

Haese, A., & Czernochowski, D. (2022). Using temporo-spatial principal component analysis as tool to dissociate latent ERP components of episodic memory retrieval: Objectifying time-window selection for overlapping ERP components. Brain and cognition, 157, 105833.

Giménez-Fernández, T., Kessel, D., Fernández-Folgueiras, U., Fondevila, S., Méndez-Bértolo, C., Aceves, N., ... & Carretié, L. (2020). Prejudice drives exogenous attention to outgroups. Social Cognitive and Affective Neuroscience, 15(6), 615-624.
